# Essential Role of Histidine for Rapid Copper(II)-Mediated Disassembly of Neurokinin B Amyloid

**DOI:** 10.3390/biom12111585

**Published:** 2022-10-28

**Authors:** Bhawantha M. Jayawardena, Lorraine Peacey, Roland Gamsjaeger, Christopher E. Jones

**Affiliations:** School of Science, Western Sydney University, Locked Bag 1797, Penrith 2751, Australia

**Keywords:** copper, neurokinin, tachykinin, amyloid

## Abstract

Neurokinin B is a tachykinin peptide involved in a diverse range of neuronal functions. It rapidly forms an amyloid, which is considered physiologically important for efficient packing into dense core secretory vesicles within hypothalamic neurons. Disassembly of the amyloid is thought to require the presence of copper ions, which interact with histidine at the third position in the peptide sequence. However, it is unclear how the histidine is involved in the amyloid structure and why copper coordination can trigger disassembly. In this work, we demonstrate that histidine contributes to the amyloid structure via π-stacking interactions with nearby phenylalanine residues. The ability of neurokinin B to form an amyloid is dependent on any aromatic residue at the third position in the sequence; however, only the presence of histidine leads to both amyloid formation and rapid copper-induced disassembly.

## 1. Introduction

Neurokinin B (NKB) is a 10-amino-acid peptide of the tachykinin family that is implicated in a range of physiological functions. Notably, NKB, acting through its receptor, NK3R, can influence reproductive health, whereby it triggers the transition to puberty and, in adults, helps maintain pulses of gonadotropin-releasing hormone (GnRH) [1]. To help regulate GnRH pulses, NKB is released from a subset of cells in the hypothalamus that also contain kisspeptin and dynorphin, and the three peptides work together to instigate and then suppress GnRH pulses [2,3,4]. Recent work has implicated NKB in thermoregulation, and studies suggest that the NKB/NK3R system is a valid therapeutic target to treat hot flushes in menopausal women [5,6,7].

Like numerous neuropeptides, NKB is stored and released from dense core vesicles (DCVs) in secretory neurons. DCVs bud from the Golgi network, mature during trafficking to the cell periphery, and then release their contents after fusion with the plasma membrane [8,9,10]. Maturation involves acidification of the vesicle and condensation of the peptide contents during which the peptides self-associate. This self-association is thought to allow the vesicles to accommodate a high concentration of peptide [11,12]. NKB and many neuropeptides found in DCVs are considered to be amyloidogenic, and the formation of the well-ordered amyloid structure contributes to high density packing [11,13]. Amyloids are fibrillar protein assemblies consisting of a cross-β-sheet structure, where the main structure contains two β-sheets, aligned perpendicular to the axis of the fiber [14]. Within β-sheets, the β-strands are held together by backbone hydrogen bonds and the amino acid side-chains can extend into the space between the sheets. The side-chains from opposing β-sheets interdigitate in the form of a ‘steric zipper’, and these interactions, along with the main-chain hydrogen bonding, help create highly stable protofibrillar structures [14]. Interactions between side-chains in the steric zipper can be diverse, but hydrophobic interactions are a common feature [11,15]. Aromatic residues are of particular interest as they have been demonstrated to be critical in the amyloidogenicity of many amyloid peptides such as amyloid β 1–42 and human islet amyloid polypeptide [16,17,18]. Aromatic residues are able to promote assembly of fibrils via π-stacking interactions, which can have favorable energetic contributions, in addition to helping to drive ordering of the fibril [19,20]. Phenylalanine (Phe) is well suited to fibril formation given that it is an aromatic and hydrophobic amino acid; consequently, it appears in the amyloidogenic sequences of several amyloidogenic peptides and proteins [19,21].

NKB (sequence = DMHDFFVGLM-NH_2_) can rapidly form an amyloid and contains a pair of Phe residues which have been proposed to support fibril formation via π-stacking interactions [22]. However, in NKB, the Phe residues themselves are not sufficient for fibril formation because mutation of histidine (His) at position 3 to either a threonine or an alanine eliminates amyloid assembly [22,23]. It is not clear how the His contributes to fibril formation given its range of possible noncovalent interactions, but it is most likely that histidine’s aromaticity and/or hydrogen bonding ability are key to fibril assembly [24,25]. The involvement of His in supporting amyloid formation underlies an important feature of NKB’s biochemistry, i.e., the ability of copper ions to rapidly disassemble a pre-formed amyloid or to inhibit assembly [22]. His is well known to coordinate copper ions, and the affinity of His for metal cations is predicted to be stronger than π–π stacking interactions [25]. Furthermore, when His is in the third position from the N-terminus, a copper coordination site termed an ATCUN motif is formed, which has a high affinity for copper ions and binds the metal via backbone nitrogen atoms and an imidazole-derived nitrogen [26]. The addition of copper to an NKB amyloid, therefore, may lead to disassembly because coordination leads to rearrangement of the His imidazole and the peptide backbone, such that the amyloid is disassembled. Physiologically, the copper-induced disassembly may be useful for generating monomeric NKB from the amyloid predicted to occur in DCVs. In this work, the role of His in NKB assembly and copper-induced disassembly is discussed.

## 2. Materials and Methods

Wildtype NKB and single-point mutants NKB(H_3_W), NKB(H_3_F), NKB(F_5_W), and NKB(F_5,6_W) were synthesized by Synpeptide (Shanghai, China) at a purity >95% and were used without further purification. Buffers were prepared from 4-ethylmorpholine (nEM), which is considered a non-coordinating buffer, or from trisaminomethane (Tris), a coordinating buffer. All water was ultrapure, metal-free MilliQ (18 MΩ·cm, Millipore, Burlington, MA, USA). CuCl_2_∙H_2_O was prepared as a 50 mM stock solution in metal-free water and diluted to appropriate working concentrations on the day of use. A 100 mM stock solution of glycine (Sigma Aldrich, Castle Hill, Sydney, Australia) was prepared in 20 mM nEM (pH 7.8). Appropriate volumes of the glycine and CuCl_2_∙H_2_O stock solutions were mixed to generate a [Cu(gly)_2_] complex. A stock solution of thioflavin T (ThT, Sigma Aldrich, Castle Hill, Australia) was prepared in metal-free water and filtered through a 0.22 μm filter (Millipore, Burlington, MA, USA). The concentration was estimated via the absorbance at 412 nm (ε = 36,000 M^−1^ cm^−1^).

### 2.1. Estimating Aggregation Propensity

Prediction of the aggregation propensity of each peptide was assessed using TANGO software [27], and mutants were presented as relative to the aggregation propensity of NKB. The conditions were 37 °C, 20 mM ionic strength at pH 7.6. The hydrophobicity of each peptide was estimated according to the grand average of hydropathicity index (GRAVY) using online calculators (e.g., gravy-calculator.de).

### 2.2. Thioflavin T Time-Resolved Fluorescence

Unless otherwise specified, 200 µM NKB, NKB(H_3_W), and NKB(H_3_F) were prepared in 20 mM nEM containing 5 µM ThT in a black-walled, clear bottom, sterile 96-well microplate (BMG Labtech, Ortenberg, Germany) at a final volume of 200 µL. The plate was covered in a transparent microplate sealing film and left to aggregate for up to 48 h at 37 °C in a microplate reader (FLUOstar OPTIMA microplate reader). ThT fluorescence was measured every 10 min through the bottom of the plate using excitation at 440 nm (30 flashes) and emission at 490 nm. The plate was automatically shaken for 5 s before each measurement. At least three replicates were included in each plate. The gain was maintained at a constant value in all experiments.

After the ThT emission of the peptides had reached a plateau, one equivalent of copper as CuCl_2_∙H_2_O or [Cu(gly)_2_] was added directly to the wells of the 96-well plate. Copper has previously been shown to not influence ThT excitation or emission [22,28,29]. The ThT emission was then recorded every 10 min for 450 min. After collection, the ThT emission of each peptide at time 0 (i.e., just prior to addition of copper) was normalized to 100% and plotted as a function of time. The data of each disassembly curve were fitted to either a mono-exponential (1) or a bi-exponential (2) decay function in Origin software (OriginLab, Northampton, MA, USA):

y = A × exp(−x/t_1_) + y0, (1)

y = A_1_ × exp(−x/t_1_) + A_2_ × exp(−x/t_2_) + y0, (2)

where y is ThT fluorescence intensity, A is amplitude, x is time, t_1_ is time constant 1, t_2_ is time constant 2, and y0 is the offset. The decay rate, k, was determined as

ki = 1/t_i_, (3)

where i = 1 or 2.

### 2.3. Tryptophan Fluorescence

Tryptophan (Trp) fluorescence was recorded on an FS5 fluorimeter (Edinburgh Instruments, Scotland, UK). The excitation wavelength was set to 280 nm, and the emission was collected over the range 300–800 nm with data points collected every 2 nm. Three scans were collected and averaged. For time-resolved Trp fluorescence of NKB(H_3_W), emission scans were collected every 5 min for 90 min.

### 2.4. Electron Microscopy

First, 200 µM NKB(H_3_W) and NKB(H_3_F) fibrils were prepared in 10 mM Tris at pH 7.4, without ThT. Then, 10 µL of each sample was aliquoted onto a 300-mesh copper formvar TEM grid and left to incubate for 10 min. The excess sample was subsequently wicked off, and the grid was negatively stained with 0.5% (*w*/*v*) uranyl acetate replacement stain (ProSciTech Pty Ltd., Townsville, Australia) for 20 s. Excess stain was wicked off, and the grid was cleaned in water for 20 s and left to dry. Images were taken with a Zeiss Merlin FEGSEM at a 30 kV acceleration voltage.

### 2.5. Mass Spectroscopy (MS)

MS data were acquired on a Xevo-ToF-1 operated in positive ion mode. The capillary voltage was 1.9 kV, and the spectra were collected at a cone voltage of 33 V and a source temperature of 80 °C over a mass range of 50 to 3000 Da. Spectra were collected and analyzed using MassLynx software (V4.1, Waters Corporation, Milford, MA, USA). The diluted samples were applied directly at an infusion rate of 10 μL per minute. Spectra were simulated in IsoPro 3.1 using natural isotopes of Cu(II) (69.17% ^63^Cu and 30.83% ^65^Cu) [30].

### 2.6. Modeling of a Two-Stranded β-Sheet

The peptide structure of a NKB antiparallel β-sheet was constructed on the basis of an existing β-sheet structure (PDB 4R8O) as a template. Two stands of NKB were constructed by replacing residues 58–67 and 71–80 of 4R8O, respectively, followed by energy-minimization performed by CNS (PMID 18007608) during which only peptide side-chains were allowed to move freely. No additional restraints were applied to any of the aromatic side-chains during energy minimizations.

## 3. Results

### 3.1. An Aromatic Residue at the Third Position in NKB Is Required for Amyloid Formation

Previous studies have shown that mutation of His3 in NKB to either a Thr [22] or to an Ala [23] abolished amyloid formation. If fibrillization is only driven by the hydrophobic C-terminal region containing the diphenylalanine sequence, then mutation of the residue at the third position should have little effect. Indeed, the algorithm TANGO showed that both NKB(H_3_T) and NKB(H_3_A) were similar to wildtype NKB in their estimated propensity to aggregate (Table 1). Alanine is a hydrophobic amino acid, and the NKB(H_3_A) mutant is more hydrophobic than wildtype NKB (Table 1), which suggests that it is the aromatic character of His that is important. To explore this further, two NKB mutants were prepared that contained either a Phe or a Trp at the third position, and these were predicted to have an aggregation propensity similar to that of wildtype NKB (Table 1).

**Figure 1:**
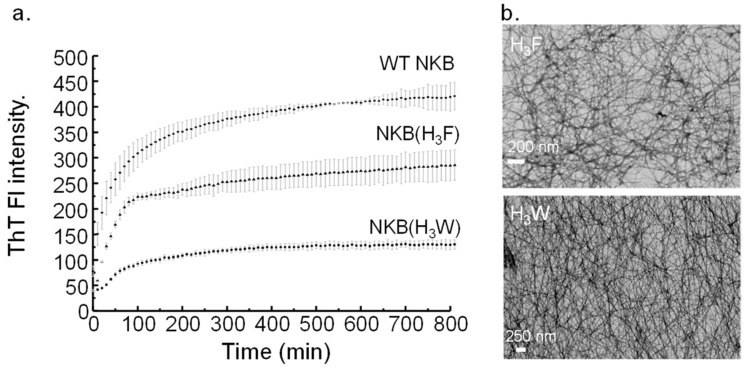
(**a**) Increasing fluorescence of ThT (ex. 440 nm, em. 490 nm) over time suggests that both NKB(H_3_F) (▲) and NKB(H_3_W) (■) generate amyloid fibrils (20 mM nEM, pH 7.7). Both mutants have lower fluorescence intensity that wildtype NKB (WT NKB (●)). Error bars represent standard deviation (N = 3). (**b**) Amyloid formation was confirmed by electron microscopy showing that both NKB(H_3_F) *top* and NKB(H_3_W) *bottom* form long unbranched fibrils.

The ability of NKB(H_3_F) and NKB(H_3_W) to form amyloid fibrils was monitored by ThT fluorescence and electron microscopy. In line with the TANGO prediction, both peptides assembled into ThT-positive fibrils (Figure 1a) with NKB(H_3_F) aggregation leading to a higher ThT fluorescence. Although this may suggest that NKB(H_3_F) forms more fibrils than NKB(H_3_W), analysis of these samples by electron microscopy showed similar fibril density (Figure 1b). It is possible that the structure of the NKB(H_3_F) fibrils allowed for greater ThT binding. Similarly, both mutants had lower ThT fluorescence intensity than wildtype NKB, which likely reflects a difference in how ThT interacts with the fibrils rather than their quantity, given that all peptides have fibrils detectable by electron microscopy [22,31]. Furthermore, the lag phase for NKB(H_3_W) was longer than for NKB(H_3_F), which may reflect the larger size of Trp compared to Phe and the time required to arrange into pre-fibrillar oligomers that were large enough to allow ThT binding. Taken together with previous data showing that NKB(H_3_T) and NKB(H_3_A) mutants do not fibrillize [22,23], this experiment supports the idea that it is aromaticity at position 3 that is important for fibrillization, and not His specifically.

### 3.2. π–π Stacking Involving the Aromatic at the Third Position in NKB Contributes to Amyloid Formation

**Figure 2:**
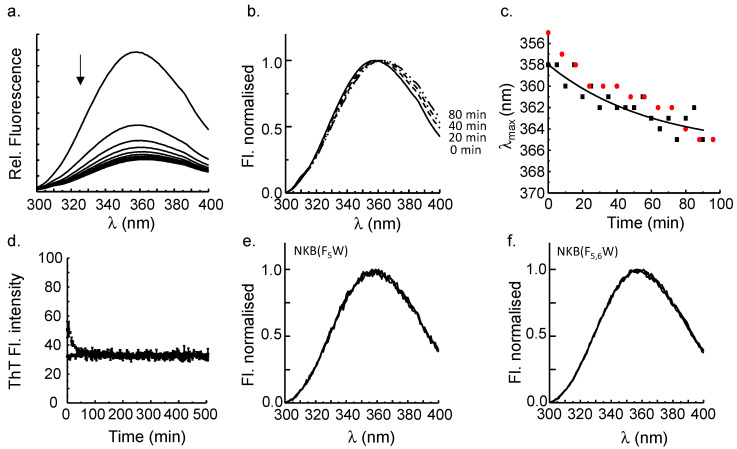
(**a**) The Trp emission (ex. 280) decreases and shifts to longer wavelengths (red shift) over time (90 min) as NKB(H_3_W) forms amyloid fibers in 20 mM nEM, pH 7.6. The arrow shows a decrease in the signal intensity during the experiment. (**b**) The normalized spectra highlight the red shift of the band maximum due to the formation of amyloid fibers. (**c**) Plotting the maximal emission wavelength (λ_max_) as a function of time shows that the wavelength shifts from 358 nm to approximately 363 nm in 90 min of amyloid formation. The red circles represent the shift observed as NKB(H_3_W) fibrillized in 20 mM Tris, pH 7.7. (**d**) NKB(F_5_W) (■) and NKB(F_5,6_W) (▲) do not show an increase in ThT fluorescence over time, suggesting that neither peptide forms fibrils. Accordingly, the Trp fluorescence maximum does not change over time for NKB(F_5_W) (**e**) or NKB(F_5,6_W) (**f**).

Having confirmed that an aromatic residue at the third position in the NKB sequence was required for amyloid formation, it was then necessary to determine if π–π stacking interactions with the aromatic residue in this position contributed to assembly. Trp fluorescence is often used to monitor changes in the environment of Trp in proteins where the emission wavelength is one property that is sensitive to local structure and dynamics [32]. In common with many aromatic systems, π–π stacking of aromatic residues generally leads to a red shift in the emission wavelength [33]. To determine if π–π stacking involving the Trp occurred in NKB, the fluorescence of the Trp in the NKB(H_3_W) mutant was monitored over time. When NKB(H_3_W) at the same concentration used for the ThT assays above (i.e., 200 μM) was monitored using Trp fluorescence, both a decrease in intensity of the emission and a red shift in the maximum wavelength (λ_max_) over time were observed (Figure 1a). The intensity loss may have been due to quenching as the Trp becomes part of the aggregated oligomers and fibrils. When the emission spectra collected over the first 80 min were normalized, the shift in λ_max_ became more apparent (Figure 2b); when λ_max_ was plotted as a function of time, it was observed that λ_max_ shifted from 358 nm to about 363 nm in 90 min (Figure 2c).

The initial λ_max_ (time 0 min) was 358 nm, which is suggestive of a highly solvent exposed Trp, as could reasonably be expected in a peptide of this size (10 residues), but this may also reflect the presence of the negatively charged aspartates adjacent to the Trp [32]. If the Trp becomes buried during fibril formation and, thus, moves from a hydrophilic to a hydrophobic environment, a blue shift of the wavelength is expected, as observed for Trp mutants of amyloid-β(1–40) [34]. The red shift observed for NKB(H_3_W) is unlikely to be due to greater solvent exposure given that we know it assembles into fibrils; thus, the shift is most likely attributed to π–π stacking interactions, which, in some cases, can lead to substantial red shifts. Although the shift was only ca. 5 nm in this experiment, it is possible that this was the mean wavelength of a blue shift as a result of solvent effects (i.e., Trp3 moved to a more hydrophobic environment in the amyloid) and a red shift due to electronic (π–π) effects. The shift did not appear to be dependent on the buffer, given that a similar trend was observed when fibrillization occurred in a Tris buffer (Figure 2c). It is worth noting, however, that the extent of the red shift was more variable than the duplicate experiments in Figure 2C suggest. It is possible that there may be some different arrangements of the aromatic residues leading to a common overall cross-β architecture. This is not unexpected given the well-known polymorphic nature of fibrils derived from many different amyloidogenic peptides and proteins [35,36,37]. To further support the idea that the red shift is due to stacking during fibrillization, NKB(F_5_W) and NKB(F_5,6_W) were analyzed, which had Phe residues changed to Trp. Although mutation to Trp retained the aromaticity, TANGO analysis (Table 1) predicted that both would have much reduced ability to aggregate. Experimentally, neither showed any ThT-positive fibril formation (Figure 2d) or detectable fibrils when viewed by electron microscopy; although this was expected from NKB(F_5,6_W), some fibril formation from NKB(F_5_W) was expected. The molecular reasons for the lack of fibril formation from NKB(F_5_W) remain to be elucidated. Nevertheless, as expected in the absence of amyloid formation, neither peptide showed any shift in maximal Trp wavelength (Figure 2e,f). Note that both peptides showed some minor loss of emission intensity (data not shown); however, it was much less than observed for NKB(H_3_W). The origin of the quenching was not investigated in this work.

### 3.3. Histidine Is Required for Rapid Copper-Induced Disassembly

**Figure 3:**
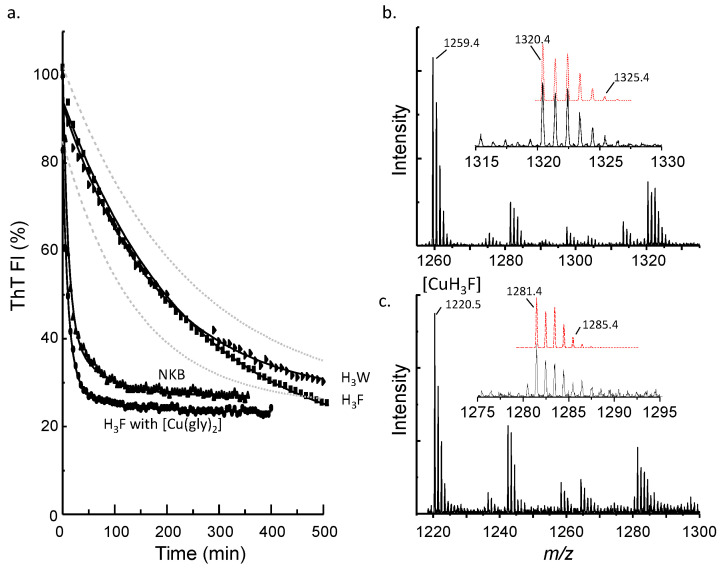
(**a**) ThT fluorescence emission intensity monitored as a function of time. Copper (one equivalent, as CuCl_2_) was added at time 0 min after previously allowing NKB (▲), NKB(H_3_F) (■), and NKB(H_3_W) (►) to aggregate in 10 mM nEM, pH 7.8 buffer. Copper can disassemble wildtype NKB faster than either NKB(H_3_F) or NKB(H_3_W). However, copper delivered as a glycine complex ([Cu(gly)_2_]) leads to rapid disassembly of NKB(H_3_F) (●) after aggregation in 10 mM nEM. Dashed lines represent the standard deviation of the NKB(H_3_W) measurement (n = 3) and are representative of the variation in all measurements. (**b**) Mass spectrum obtained after copper addition to NKB(H_3_W) shows peaks due to metal-free peptide (*m/z* 1259.4) and to metal-bound peptide (*m/z* 1320.4). (**c**) Mass spectrum obtained after copper addition to NKB(H_3_F) shows peaks due to metal-free peptide (*m/z* 1220.5) and to metal-bound peptide (*m/z* 1281.4). In both panels (**b**,**c**), the inset highlights the peak manifolds corresponding to copper-bound peptides with the simulated spectrum (red dotted line) assuming one copper ion is bound to the peptide.

Previous studies have shown that the copper disassembly of NKB fibrils can be monitored using ThT fluorescence. Disassembly leads to a loss of ThT fluorescence, which has been corroborated by analysis using atomic force microscopy [23] and electron microscopy [22]. Firstly, the wildtype NKB was analyzed. After allowing NKB to fibrillize the rate at which Cu (equimolar, as CuCl_2_∙×H_2_O) could disassemble, the amyloid was best fitted using a bi-exponential decay to the ThT data (Figure 3a), which gave decay rates of 9.8 × 10^−2^ min^−1^ and 1.5 × 10^−2^ min^−1^. These data suggest that more than one mechanism contributes to copper-induced fibril disassembly. Next, one equivalent of copper was added to ThT-positive fibrils of NKB(H_3_W) and NKB(H_3_F), and the fluorescence was monitored over time. Both peptides showed a slow loss of ThT intensity over ~7.5 h (Figure 3a), which can reasonably be interpreted as slow disassembly of the peptides. In contrast to the disassembly of NKB, these plots were best fit with a mono-exponential decay function, and both had similar decay rates (NKB(H_3_F) = 4.2 × 10^−3^ min^−1^; NKB(H_3_W) = 5.5 × 10^−3^ min^−1^). These peptides were ~3.5× slower than the slowest wildtype NKB rate of disassembly in the presence of copper. It was surprising that copper could disassemble the NKB(H_3_W) and NKB(H_3_F) amyloids at all, but it was surmised that the reason was because Cu^2+^ ions could still coordinate to the peptide N-terminus and amide nitrogen atoms to alter the structure of the peptide backbone. To determine if NKB(H_3_W) and NKB(H_3_F) were coordinating metal, mass spectroscopy was used to analyze the samples after copper had disassembled the fibrils. The mass spectrum of NKB(H_3_W) showed predominant peaks due to copper-free peptide (*m/z* 1259.4) (Figure 3b). However, a significant peak at *m/z* 1320.4 was attributed to copper-bound NKB(H_3_W). Simulation of this peak manifold assuming a single copper bound to the peptide with loss of three amide protons (i.e., [H_3_W + Cu(II) − 3H]^+^, pred. *m/z* 1320.48) is in excellent agreement with the experimental spectrum (Figure 3b, inset). Similarly, the mass spectrum for NKB(H_3_F) showed a predominant peak at *m/z* 1220.5 from metal-free NKB(H_3_F) and a peak at *m/z* 1281.4 attributable to copper-bound NKB(H_3_F) (Figure 3c). Simulation of the peak manifold at *m/z* 1281.4 assuming a single copper bound to NKB(H_3_F) ([H_3_F + Cu(II) − 3H]^+^, pred. *m/z* 1281.0) resulted in a peak distribution that matched the experimental spectrum very well (Figure 3c, inset). The presence of metal-free peptide in the mass spectra suggests that copper does not need to bind to all peptides to disrupt the fibril structure or, more likely, that some copper is lost during the electrospray ionization process.

Given that wildtype NKB, NKB(H_3_W), and NKB(H_3_F) all bind copper ions, the difference in fibril disassembly rates must lie in the coordination environment and the resulting affinity for copper. NKB is thought to coordinate via the His nitrogen, two amide nitrogens, and the N-terminal nitrogen to form a 4N, square-planar environment, although this may be altered in membrane or lipid environments [23,38,39,40]. In contrast, at the pH used here (~pH 7.8), the mass spectra suggest that both NKB(H_3_W) and NKB(H_3_F) likely bind to the N-terminal nitrogen and two amide nitrogens ({NH_2_, 2N^−^}) resulting in the loss of three protons. The fourth coordination site can reasonably be suggested to be taken by a solvent molecule, most likely water. When the addition of copper ions to NKB(H_3_W) is monitored by electronic spectroscopy, a peak at ~625 nm is observed (data not shown) which is consistent with copper ions coordinated with nitrogen and oxygen atoms [41]. The copper site of the type formed by NKB is considered a higher affinity site than that formed in NKB(H_3_W) and NKB(H_3_F), which is attributable to the chelate rings formed and the presence of a strong-field nitrogen donor in the fourth position in NKB compared to NKB(H_3_W) and NKB(H_3_F). To explore the notion that supporting copper binding to a non-His peptide by including an additional strong-field ligand may lead to more rapid fibril disassembly, copper as a glycine complex ([Cu(gly)_2_]) was added to fibrils of NKB(H_3_F). In contrast to the addition of Cu(II) as CuCl_2_, addition as the glycine complex resulted in a rapid loss of ThT fluorescence intensity (Figure 3a). The decay was best fitted using a bi-exponential decay function to give fast and slow rates of 9.4 × 10^−2^ min^−1^ and 1.2 × 10^−2^ min^−1^, respectively, which are similar to that of NKB in the presence of CuCl_2_. It is predicted that a ternary [Cu(H_3_F)(gly)] complex is formed, with glycine displacing the solvent molecule as a copper ligand, which helps drive fibril disassembly. To ensure that [Cu(gly)_2_] itself was not disassembling the NKB(H_3_F) fibrils, the non-coordinating nEM buffer was substituted for a Tris buffer. Tris is well known to be a coordinating buffer which can potentially contribute to the formation of ternary complexes [42,43]. NKB(H_3_F) in this buffer readily formed ThT-positive fibrils, and the addition of copper (as CuCl_2_) resulted in a rapid loss of intensity with a bi-exponential mechanism and rates similar to those observed with copper addition to NKB (data not shown).

## 4. Discussion

Amyloid fibrils are considered stable protein assemblies that can resist disassembly, as exemplified in diseases such as Alzheimer’s. As noted by Maji et al. (2009), this concept poses challenges for peptides packed into dense core vesicles as an amyloid, given the structure needs to disassemble upon release so that the monomeric peptide can interact with its receptor. However, release of monomers from amyloids has been shown, and it has been suggested that dilution effects and the presence of different components in the extracellular environment can lead to disassembly [13,44]. The formation of an amyloid by NKB is an important feature that underlies not only its packaging into secretory vesicles but also its ability to co-assemble with amyloid-β(1–40) [45,46]. Disassembly of NKB amyloid occurs upon copper coordination, and the presence of this metal in the medium can also inhibit assembly [22]. Copper ions coordinate to the single His at sequence position 3, and it has been suggested that this coordination disrupts the involvement of His in an aromatic cluster. This has support because mutation of the His to a nonaromatic but hydrophobic [23] or hydrophilic [22] amino acid abrogated amyloid formation, but mutation to an aromatic (Trp or Phe) led to robust fibril formation. This clearly indicates that it is the aromatic character of His that is required for amyloidogenesis in the wildtype peptide. It is likely that His forms an aromatic cluster with the Phe residues at the fifth and sixth positions in the NKB sequence. This is supported by the fluorescence experiments with a mutant peptide that contains Trp at the third position that shows a time-dependent red shift in emission that correlates with formation of the fibril and suggests a π–π stacking interaction. Intriguingly, the fitting of a bi-exponential function to the disassembly of the wildtype NKB fibril in the presence of copper ions suggests that at least two mechanisms account for the disassembly. It is reasonable to speculate that coordination of copper to the peptide is one step, and break-up of the aromatic cluster represents another, slower step. In the mutants NKB(H_3_F) and NKB(H_3_W), the high-affinity binding step is not present, and the disassembly data can be fitted assuming a single mechanism that encompasses low-affinity binding and aromatic cluster breakup. Nevertheless, the mechanisms leading to copper-induced disassembly require further investigation.

**Figure 4:**
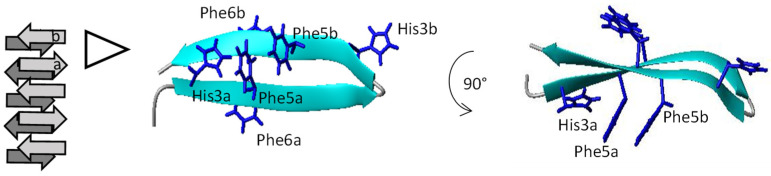
Energy minimization of two strands from one β-sheet in an NKB fibril (cartoon of fibril shown on left) highlights how His can interact with the Phe residues via π–π contacts. His3 and Phe5 in one strand (strand a) interact with Phe5 in strand b. Energetically favorable edge-to-face and offset face-to-face contacts are observed in this model.

To model how His might interact with the Phe residues to form an aromatic cluster, the program CNS was used to energy minimize two strands of a fibril β-sheet where each strand was NKB (Figure 4). The structure was energy-minimized using only backbone hydrogen bonds as constraints, and the side-chains were left unconstrained. In this model, a cluster involving His3 and Phe5 from one strand (Figure 4, strand a) associates with Phe5 from the other strand (strand b), where the two Phe amino acids adopt an offset, stacked arrangement with the His in an edge-to-face arrangement with Phe5 (Figure 4). These arrangements are considered favorable and often observed in aromatic clusters, as well as in intra-sheet aromatic interactions in amyloids [47,48]. If the edge-to-face interaction observed for His3 is retained when His is mutated to either Phe or Trp, then fibril formation would still occur, as observed experimentally.

In the absence of experimental structural data for the NKB amyloid, it is difficult to establish how or if His3 is involved in inter-sheet interactions, but the model does provide clues to how copper may promote fibril disassembly. Copper coordinates to a His imidazole nitrogen and the amide nitrogen. This coordination can pull the His side-chain away from the aromatic cluster, causing disruption of the intra-sheet aromatic cluster, destabilization of the amyloid, and rapid disassembly. The mutant peptides NKB(H_3_F) and NKB(H_3_W) slowly disassemble in the presence of copper ions because, although the metal does not coordinate to the aromatic side-chain, it can still interact with the backbone nitrogen atoms. Supporting the generation of higher-affinity complexes with the mutant peptides by supplying an additional coordinating molecule does lead to rapid disassembly that follows a similar two-step mechanism to wildtype NKB. Ternary complexes often have a higher metal affinity than the peptide alone [49]. Overall, the data presented here suggest that, in NKB, amyloid formation is not dependent on the presence of His but requires an aromatic residue at the third position in the sequence. However, with His at the third position, the presence of copper promotes rapid amyloid disassembly because of side-chain and backbone coordination. Of all the biological metals, copper is uniquely capable of this type of coordination, and this leads to disruption of the His aromatic cluster. Physiologically, cells only need to regulate the presence of extracellular copper in order to disassemble NKB amyloids released from dense core vesicles.

## 5. Conclusions

Often, the role of His as a molecular switch in protein structures, including ordered stuctures such as nanotubes, is only linked to pH-induced changes given that the pKa of the imidazole side-chain is within a physiological range [50]. The work described here highlights a copper-dependent mechanism for regulating the assembly and disassembly of amyoids. This activity is dependent on the coordination ability of His and provides insight into how coordination chemistry can be exploited to regulate formation of functional nanomaterials.

## Figures and Tables

**Table 1:** Sequences, hydrophobicity, and aggregation propensity of wildtype NKB and mutants.

Name	Sequence	HydroP ^1^	%Agg ^2^
NKB	DMHDFFVGLM-NH_2_	0.68	100
H_3_T	DMTDFFVGLM-NH_2_	0.93	93.2
H_3_A	DMADFFVGLM-NH_2_	1.18	93.1
H_3_W	DMWDFFVGLM-NH_2_	0.91	93.3
H_3_F	DMFDFFVGLM-NH_2_	1.28	93.4
F_5_W	DMHDWFVGLM-NH_2_	0.31	59.5
F_5,6_W	DMHDWWVGLM-NH_2_	0.30	26.7

^1^ Hydrophobicity (GRAVY). ^2^ TANGO-predicted aggregation normalized to NKB propensity.

## Data Availability

Datasets generated and/or used in this study are available from the corresponding author upon reasonable request.
